# A Selective Review and Virtual Screening Analysis of Natural Product Inhibitors of the NLRP3 Inflammasome

**DOI:** 10.3390/molecules27196213

**Published:** 2022-09-21

**Authors:** Sherihan El-Sayed, Sally Freeman, Richard A. Bryce

**Affiliations:** 1 Division of Pharmacy and Optometry, School of Health Sciences, Manchester Academic Health Sciences Centre, University of Manchester, Oxford Road, Manchester M13 9PT, UK; 2 Department of Medicinal Chemistry, Faculty of Pharmacy, Zagazig University, Zagazig 44519, Egypt

**Keywords:** NLRP3, IL-1β, inflammation, natural products, docking, design

## Abstract

The NLRP3 inflammasome is currently an exciting target for drug discovery due to its role in various inflammatory diseases; however, to date, no NLRP3 inhibitors have reached the clinic. Several studies have used natural products as hit compounds to facilitate the design of novel selective NLRP3 inhibitors. Here, we review selected natural products reported in the literature as NLRP3 inhibitors, with a particular focus on those targeting gout. To complement this survey, we also report a virtual screen of the ZINC20 natural product database, predicting favored chemical features that can aid in the design of novel small molecule NLRP3 inhibitors.

## 1. Introduction

**Figure 1:**
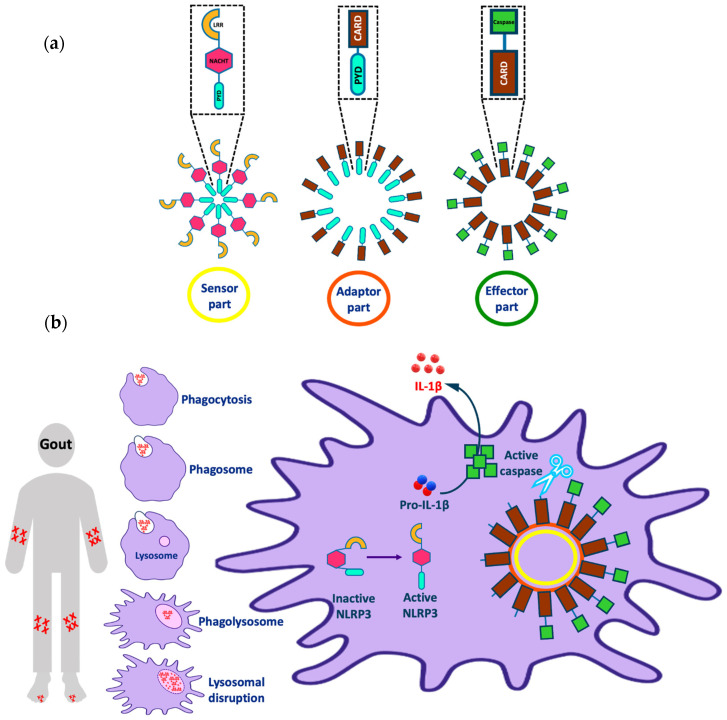
Representation of (**a**) different parts of the NLRP3 inflammasome and (**b**) the activation of the NLRP3 inflammasome by uric acid and implications in gout. Note that this figure was modified from Figure 1 in our recent publication [4].

The NLRP3 (NOD-, LRR-, and pyrin domain containing 3) inflammasome is one of the most interesting targets implicated in various inflammatory diseases (e.g., Alzheimer’s disease, atherosclerosis, and gout). The inflammasome consists of three parts: a sensor molecule, adaptor protein, and effector (Figure 1a). The sensor part of the inflammasome is the PRR (pattern recognition receptor) that triggers inflammasome assembly in response to DAMPs (damage-associated molecular patterns) or PAMPs (pathogen-associated molecular patterns). PRRs can be classified into two main classes: the NOD (nucleotide-binding and oligomerization domain-like receptor (NLR)) family, including, for example, the protein NLRP3; and the non-NLR family, which has members such as the protein AIM2 (absent in melanoma 2). AIM2 can bind directly to the stimulus via the HIN (hemopoietic expression-interferon inducibility-nuclear localization) domain; however, NLRP3 is activated indirectly in response to various stimuli [1]. The adaptor protein is referred to as ASC (apoptosis-associated Speck-like protein containing a CARD) and it consists of a pyrin domain, which binds to the sensor protein via pyrin–pyrin interactions; and a CARD (caspase recruitment and activation) domain, which binds to procaspase-1 via CARD–CARD interactions. The effector is a protease caspase-1 that is responsible for cytokine activation and pyroptosis [1,2].

The NLRP3 inflammasome is a cytoplasmic macromolecule often present in macrophages that regulate the activation of potent inflammatory mediators and is implicated in the pathogenesis of numerous non-infectious diseases. NLRP3 consists of three domains: an N-terminal pyrin (PYD) domain, which binds to ASC; a central adenosine triphosphatase NACHT domain; and a C-terminal leucine-rich repeat (LRR) domain [3]. Although NLRP3 can respond to a wide range of stimuli other than pathogenic molecules, the mechanism of NLRP3 activation has not been fully characterized; there are several theories describing this activation [4]. In many cells, NLRP3 activation passes through two stages: priming and activation. In normal cells, the amount of NLRP3 is insufficient to activate inflammasome assembly, so a priming stage is required, which involves overexpression of the NLRP3 inflammasome components through the activation of the transcription factor NF-κB. The activation of NF-κB can be achieved through various stimuli, for example, the binding of PAMPs such as lipopolysaccharide (LPS) to the membrane-bound receptor TLR4 (toll-like receptor 4). Then, NLRP3 activation occurs through either ion-dependent or ion-independent pathways [5,6,7].

### 1.1. Ion-Dependent Activation Pathways

High levels of extracellular ATP, which bind to the P2X purinoceptor 7 (P2X7) channel, increase cell membrane permeability to potassium, which can activate NLRP3 in primed cells. As an example of stimulated inflammation, crystal accumulation due to pathological conditions (e.g., cholesterol, uric acid), foreign inhaled crystals (e.g., silica, asbestos), and proteinaceous aggregates (e.g., amyloid-β) are phagocytosed by lysosome and lead to lysosomal disruption. Cathepsins released after lysosomal damage increase K^+^ efflux via an ATP-dependent mechanism [5,8]. Some studies have shown that chloride efflux via volume-regulated anion channels (VRACs) can also trigger NLRP3 activation [9,10]. In addition, mitochondrial stress and increased calcium influx contribute to increased intracellular reactive oxygen species (ROS), which activate NLRP3 [11].

### 1.2. Ion-Independent Activation Pathways

Inhibition of glycolysis, inhibition of mitochondrial NADH oxidase, and displacement of hexokinase 2 from mitochondria have been linked to NLRP3 activation via an ion-independent mechanism [5]. Once activated, NLRP3 and ASC move from the endoplasmic reticulum and mitochondria, respectively, to form the inflammasome complex in the cytoplasm. The NLRP3 NACHT domain promotes oligomerization of the NLRP3 pyrin domain to bind ASC via pyrin–pyrin interactions. ASC is then converted to a prion-like form and long ASC specks or pyroptosome are produced, which play an important role in NLRP3 activation. Pro-caspase-1 then binds to ASC via CARD–CARD interactions and forms its own prion-like filaments that branch off the ASC filaments. Procaspase-1 consists of two fragments: a p35 fragment, which contains both a CARD domain and p20 subunit, and a p10 fragment. Active caspase-1 is formed after heterodimerization of two molecules of p20 with two molecules of p10. Active caspase-1 then activates pro-IL-1β by its conversion to IL-1β, which is released from the cell and causes tissue damage and/or repair [12,13]. 

Recently, studies reported that NEK7 (NIMA-related kinase 7), a member of the NIMA (‘never in mitosis gene A’)-related serine-threonine kinase family, plays a key role in NLRP3 activation. Its binding to the C-terminal LRR domain of NLRP3 during interphase is pivotal for NLRP3-ASC-caspase 1 assembly. NEK7 is a mitotic kinase, which also serves in mitotic spindle formation and centrosome separation in the cell cycle. The amount of NEK7 in macrophages is insufficient to enable its dual action simultaneously; thus, NEK7 activates NLRP3 only in interphase. Interestingly, NLRP3-NEK7 binding is linked to potassium efflux, although the exact mechanism is unknown [3,13,14]. 

Despite its role in the defense against invading pathogens and in tissue repair, NLRP3 inflammasome activation is implicated in the pathogenesis of a range of serious conditions. Inflammasome-dependent diseases include cancer, metabolic disorders (e.g., type 2 diabetes), diseases caused by the accumulation of crystals (e.g., gout or atherosclerosis), and diseases caused by the formation of protein aggregates (e.g., Alzheimer’s disease) [15,16,17,18]. Considering in more detail the problem of gout as an example (Figure 1b), we observe that this disease is characterized by the deposition of monosodium urate (MSU) crystals in the joints when its plasma concentration is >420 μM, leading to joint swelling and inflammation. Crystal accumulation activates the immune system, activating the macrophage to remove the accumulated crystals by phagocytosis to form a phagosome. The phagosome fuses with the lysosome in the cytoplasm of the macrophage to form a phagolysosome. Further crystal accumulation in the phagosome leads to lysosomal disruption that activates the sensor part of the NLRP3 inflammasome from its inactive closed form to an active open form. After, the active NLRP3 sensor oligomerizes and binds to the adaptor and effector parts of the inflammasome as mentioned earlier. Finally, this process leads to the release of active IL-1β from the cell, which causes an inflammatory effect and joint pain in gout patients [18,19,20,21,22,23,24].

### 1.3. MCC950 and Analogues as Inhibitors of NLRP3

**Figure 2:**
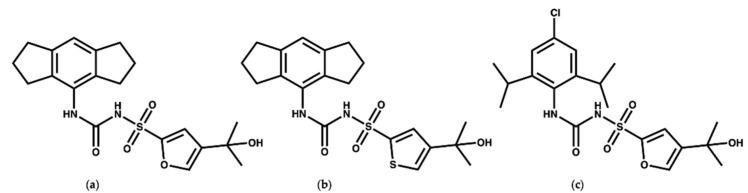
Chemical structures of (**a**) MCC950, (**b**) the thiophene isostere of MCC950 [24], and (**c**) NP3-146.

MCC950 (Figure 2a) is a diaryl sulfonyl urea derivative known as cytokine release inhibitory drug 3 (CRID3). MCC950 is considered as the most potent and selective NLRP3 inhibitor to date, with an *IC_50_* of 7.5 nM in mouse bone marrow-derived macrophages (BMDMs) and an *IC_50_* of 8.1 nM in human monocyte-derived macrophages (HMDMs) [25,26]. Mechanistic studies revealed that MCC950 binds to the NLRP3^NACHT^ subdomains, hindering the ATPase activity, driving NLRP3 into the inactive closed conformer [27,28,29]. Moreover, MCC950 can bind to both active (open) and inactive (closed) conformers of NLRP3 [30,31]. Although MCC950 is one of the most potent direct selective inhibitors of NLRP3, the reported renal and hepatic toxicity restricts its therapeutic development, which may be attributed to the furan moiety of MCC950 [32,33]. Therefore, it is essential to develop potent NLRP3 inhibitors with a new chemical scaffold. 

Keuler and coworkers [34] studied the chemical stability of a series of 12 sulfonyl urea analogues of MCC950 using HPLC, and the affinity of the compounds with NLRP3 was also determined using surface plasmon resonance spectroscopy. Their study revealed that the anionic form of MCC950 and its analogues are more stable than the neutral form. Additionally, Keuler and coworkers reported that the thiophene isostere (Figure 2b) had the same potency and stability as MCC950, which could be of use for the future design of novel inhibitors. Interestingly, their study suggested that the tertiary alcohol group is important both for the chemical stability and activity of the MCC950 analogues [34]. In 2021, a crystal structure of the NACHT domain in complex with NP3-146 (Figure 2c) was solved by Dekker and coworkers [35] (PDB code 7ALV, resolution 2.8 Å). The fluorescent probe NP3-146 inhibits the NLRP3 activity and the release of IL-1β at a concentration of 20 nM [35]. 

In this piece of work, we started with a short introduction to the NLRP3 inflammasome activation process and its implication in inflammatory diseases. Then, selected natural products reported in the literature as NLRP3 inhibitors will be discussed, with a particular focus in some which target gout. Finally, a virtual screen (VS) of the ZINC20 natural product database was performed to provide insight into the future design of selective NLRP3 inhibitors.

## 2. Reported NLRP3 Natural Product Inhibitors

Inflammasomes play an important role in the pathogenesis and progression of diseases so they are considered as important therapeutic targets. Inflammasome inhibitors have the potential to treat a number of life-threatening diseases. There are different suggested mechanisms for the inhibition of inflammasome activity. Here, we provide selected examples of natural products (NPs) that have been reported in the literature as NLRP3 inhibitors, with a focus on those relating to gout. For NPs targeting other diseases, we direct the reader elsewhere [36,37,38,39,40,41,42,43,44,45].

### 2.1. Glycyrrhizin and Isoliquirtigenin

**Figure 3:**
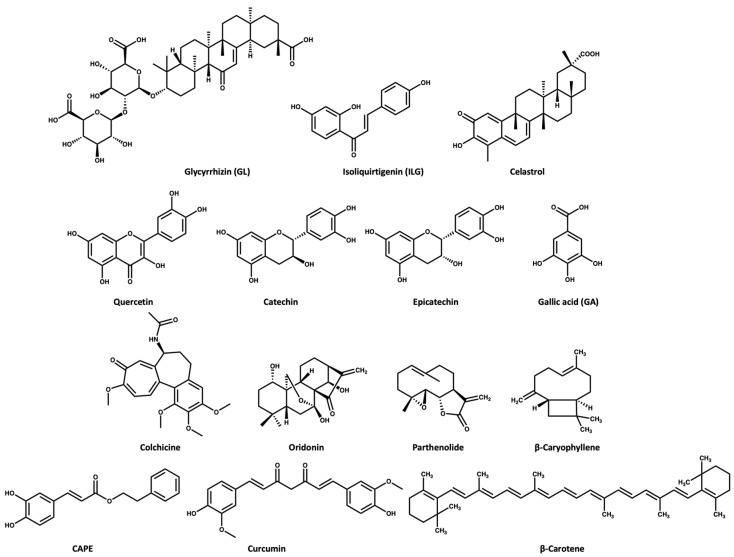
Chemical structures of the selected NPs that are known inhibitors of the NLRP3 inflammasome.

ASC oligomerization is a key step in inflammasome activation. Glycyrrhizin (GL) and isoliquirtigenin (ILG) (Figure 3)are flavonoid derivatives from Glycyrrhiza uralensis that exert their inflammasome inhibitory activity either by inhibiting ASC pyroptosome formation or LPS- NF-κB activation [46]. GL can inhibit both NLRP3 and AIM2 inflammasome activation while ILG is a selective inhibitor for NLRP3 inflammasome with an *IC_50_* value of 10.1 μM [46,47]. In the study conducted by Hiroe and coworkers [46], the IL-1β release was 3 ng/mL in the ATP-induced NLRP3 inflammasome activation when the cells were treated with 1000 and 1 μM of GL and ILG, respectively. This indicates that ILG is more potent than GL. Moreover, ILG can inhibit IL-1β release in response to MSU-induced NLRP3 activation at a concentration of 10 μM.

### 2.2. Celastrol

Celastrol (Figure 3) triterpenoid isolated from the roots of *Tripterygium wilfordii*, shows a promising anti-inflammatory effect. The Chinese National Medical Products Administration has approved the use of *Tripterygium wilfordii* tablets for the treatment of rheumatoid arthritis: a concentration of 25–50 nM of celastrol inhibits the IL-1β release in ATP- and LPS-induced NLRP3 activation [48]. Yu and coworkers [49] reported that a dose of 125 nM of celastrol could inhibit the IL-1β release in vivo and in vitro through preventing the oligomerization of ASC and subsequently NLRP3 activity [49].

### 2.3. Quercetin and Procyanidins 

Quercetin is a dietary flavonoid (Figure 3) present in many fruits and vegetables as glycosides, ethers, or sulfates. Quercetin is known to have beneficial effects on human health [50,51,52]. Quercetin has been reported to show an anti-inflammatory effect in gouty arthritis caused by MSU, showing inhibition of IL-1β release at a concentration of 30 μM, with reports suggesting that this effect is related to inhibition of the NLRP3 inflammasome [53,54]. Procyanidins are a group of flavonoids that exist as secondary metabolites in fruits (e.g., cherries, grapes). Procyanidins exist in two forms: monomer (catechin and epicatechin (Figure 3), and homopolymer [55,56]. Several studies suggest that the intake of procyanidins (or consuming cherries) helps to decrease joint swelling and pain associated with gout. It has also been reported that procyanidins at a concentration of 10 μM can inhibit NLRP3 activation induced by MSU in vitro [57].

**Figure 4:**
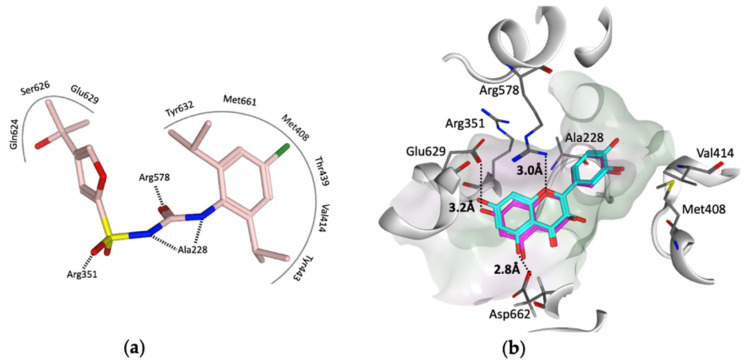
(**a**) Representation of the MCC950–analogue (NP3-146) interactions from the NACHT domain X-ray structure (PDB code 7ALV) [25]. (**b**) Docked poses of quercetin (cyan) and epicatechin (magenta) in the ligand binding site of the NACHT domain. Black dotted lines represent the hydrogen bonds, and the pocket is colored based on the lipophilicity; polar surface (purple) and nonpolar surface (green).

To provide insight into the potential interaction of quercetin and the procyanidins monomers (catechin and epicatechin) with NLRP3, here, we docked each compound in turn into the cofactor site and the known (MCC950) inhibitor binding site in the NACHT domain of NLRP3 [35] using the software OEdocking [58]. Quercetin and the procyanidins did not dock well into the cofactor site; however, quercetin and epicatechin were both a good fit for the inhibitor binding site, with chemgauss4 docking scores of −10.8 and −10.5, respectively (Figure 4). The oxygen atom of the chromen-4-one ring in quercetin and the chroman ring in epicatechin form hydrogen bonds with Arg578, which is one of the key residues for MCC950 binding observed in the crystal and cryo-EM structures (Figure 4a). Moreover, two hydrogen bonds formed between the hydroxyl groups of the ligands and the carboxylate side chain of Glu629 and Asp662 (Figure 4b). This suggests that these polyphenols may be used as lead compounds to design novel selective inhibitors of the NLRP3 inflammasome.

### 2.4. Gallic Acid

Gallic acid (GA, Figure 3) belongs to the polyphenol class of phytochemicals and is widely distributed in plants such as pomegranates, guava, mulberry, and tea leaves. GA is known for its antioxidant and anti-inflammatory activity [59,60]. A study revealed that GA has anti-inflammatory activity in gouty arthritis due to NLRP3 inhibition. Their study suggested that GA at a concentration of 80 μM can inhibit MSU-mediated NLRP3 activation and inflammasome oligomerization via inhibition of NEK7 binding to NLRP3 in vitro [61]. Moreover, a dose of 100 mg/kg GA was effective in treating knee joint swelling in a mice model by inhibiting the IL-1β release compared to a dose of 1 mg/kg for colchicine [61].

### 2.5. Colchicine 

The *Colchicum autumnale* plant, also known as the autumn crocus, is the natural source of colchicine (Figure 3) Colchicine is a microtubule inhibitor [62], which has been used for the treatment of gout since ancient Egyptian times, receiving approval by the FDA in 2009 [62,63,64]. Bonaventura and coworkers [65] recently reported that the anti-inflammatory effect of colchicine is related to its ability to inhibit NLRP3 inflammasome oligomerization, which subsequently causes inhibition of the release of cytokines. However, the exact mechanism is unclear, with a suggestion that the microtubule depolymerization by colchicine in immune cells may negatively affect inflammasome oligomerization [65,66].

### 2.6. Oridonin

**Figure 5:**
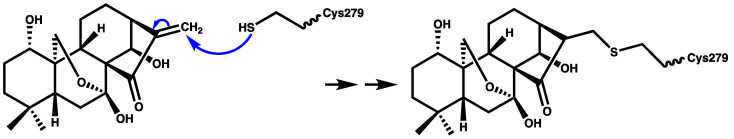
Schematic representation of covalent bond formation between oridonin and cysteine 279 of the NACHT domain.

Oridonin (Figure 3) is a herbal medicine used for the treatment of inflammatory diseases, for example, gout, peritonitis, and type-2 diabetes. Oridonin’s anti-inflammatory activity is due to its covalent binding to cysteine 279 of NACHT through a Michael addition, which prevents the NEK7–NLRP3 interaction (Figure 5). Oridonin inhibits NLRP3 activity with an *IC_50_* value of 0.75 μM [67,68].

### 2.7. Parthenolide 

Parthenolide (Figure 3) belongs to the sesquiterpene lactone phytochemical class, which is widely used for the treatment of inflammatory disorders. It has been reported that the anti-inflammatory effect of parthenolide is due to its binding to cysteine residues of caspase-1 so it can inhibit inflammasomes and subsequently cytokine release [69,70]. Juliana and coworkers [70] reported that parthenolide at a concentration of 10 μM can selectively inhibit the ATPase activity of NLRP3 through binding to cysteine in the p20 subunit of caspase-1. In addition, we propose that the mechanism of parthenolide binding to cysteine of caspase-1 may be similar to the oridonin binding to the cysteine residue of NACHT (Figure 5) due to the structural similarity between parthenolide and oridonin.

### 2.8. β-Caryophyllene 

β-Caryophyllene (Figure 3) is a bicyclic sesquiterpene that is most abundant in the essential oils extracted from oregano, cinnamon, rosemary, thyme, basil, mint, cloves, and ginger. β-Caryophyllene shows various biological activities through its binding to cannabinoid receptors [71,72]. Recently, Li and coworkers [73] reported that β-caryophyllene can block the MSU-induced activation of NLRP3 in vivo using a dose of 100 mg/kg. Moreover, their study suggests that the mechanism of action of β-caryophyllene may be through direct binding to NLRP3 or indirect inhibition of NF-κB, caspase-1, ASC, and/or TLR4 [73].

### 2.9. CAPE

Caffeic acid phenethyl ester (CAPE) (Figure 3) naturally extracted from propolis (a resin made by bees), is proposed to be effective for the treatment of acute gout [74]. CAPE is a small molecule that shows NLRP3 inhibitory activity at a concentration of 10 μM. CAPE exerts its inhibitory activity via direct binding to ASC^PYD^ but not NLRP3^PYD^, thus preventing ASC-NLRP3 oligomerization [74].

### 2.10. Curcumin

Curcumin (Figure 3) extracted from turmeric (*Curcuma longa*), is used widely as a herbal supplement due to its antioxidant and anti-inflammatory activities [75,76]. Studies have shown that the role of curcumin in inflammatory diseases may be attributed to its inhibitory effect on the NF-κB signaling pathway, which is involved in inflammasome activation [77,78]. Poor water solubility, fast metabolism at physiological pH, and poor bioavailability of curcumin are the main challenges in studying its therapeutic effect; however, it is noted that piperine increases its bioavailability [79]. To overcome these limitations, studies have used metals or polypeptides as a delivery system to improve the solubility and bioavailability of curcumin [78,80]. Zhang and coworkers [78] used tetrahedral framework nucleic acids (TFNAs) as a carrier for curcumin to improve its bioavailability. Then, in vivo testing of the curcumin–TFNAs complex using a mouse model of gout induced by MSU revealed that the complex can manage joint swelling at a concentration of 40 μM.

### 2.11. β-Carotene 

β-Carotene (Figure 3) is a prodrug of retinol (vitamin A) that exists in most fruit and vegetables. In 2020, Yang and coworkers [81] studied the NLRP3 inhibitory activity of β-carotene using gout as a disease model. This study revealed that β-carotene (30 mg/kg in vivo and 20 μM in vitro) can selectively inhibit NLRP3 through its direct binding to the pyrin domain. Of note, oral administration of β-carotene was of benefit in the treatment of gouty arthritis in mice [69,81].

## 3. Virtual Screening to Identify Possible Natural Product Scaffolds Targeting NLRP3 

**Figure 6:**
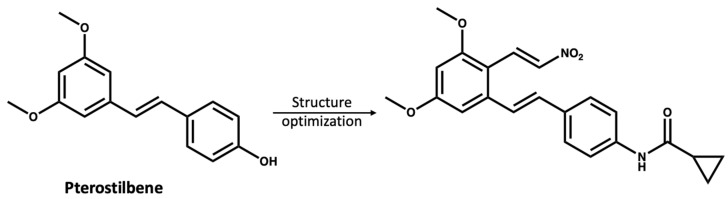
The reported [87] structure optimization of pterostilbene, leading to the discovery of a more potent NLRP3 inhibitor.

The study of NPs as treatments for various diseases is of substantial interest to the scientific community; however, there are limitations as many NPs are present at low concentrations in the natural source and have complex structures, making their synthesis challenging [82,83]. Moreover, most NPs are non-selective for certain protein targets and need a high dose to have a therapeutic effect [84]. However, compounds from natural sources could be used as inspiring lead compounds to rationally design novel selective small molecules [85,86,87]. Chen and coworkers [87] used pterostilbene (*IC_50_* > 10 mM), which was extracted from blueberries as a lead compound to develop a more potent NLRP3 inhibitor with an *IC_50_* value of 0.56 µM (Figure 6).

**Figure 7:**
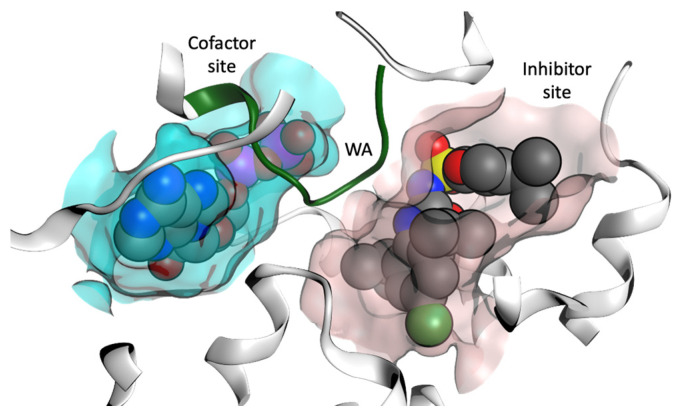
Representation of the cofactor (ADP) site and the inhibitor binding site from the X-ray structure of the NACHT domain of NLRP3 (PDB code 7ALV) [25]. Walker A (WA) site is colored green.

In order to identify NP scaffolds that could provide potent specific interactions with NLRP3, in this current work, we performed VS of 100,000 compounds from the ZINC20 natural products database (https://zinc20.docking.org (accessed on 15 August 2022)) using the OpenEye [58] software suite. This subset of ZINC20 was selected according to physical properties (Table S1), then docked into the MCC950 inhibitor binding site and the ADP cofactor site from the crystal structure of the NLRP3-NACHT domain (Figure 7, PDB code 7ALV) [35]. A final selection of compounds for the cofactor site and the inhibitor site are discussed. 

**Figure 8:**
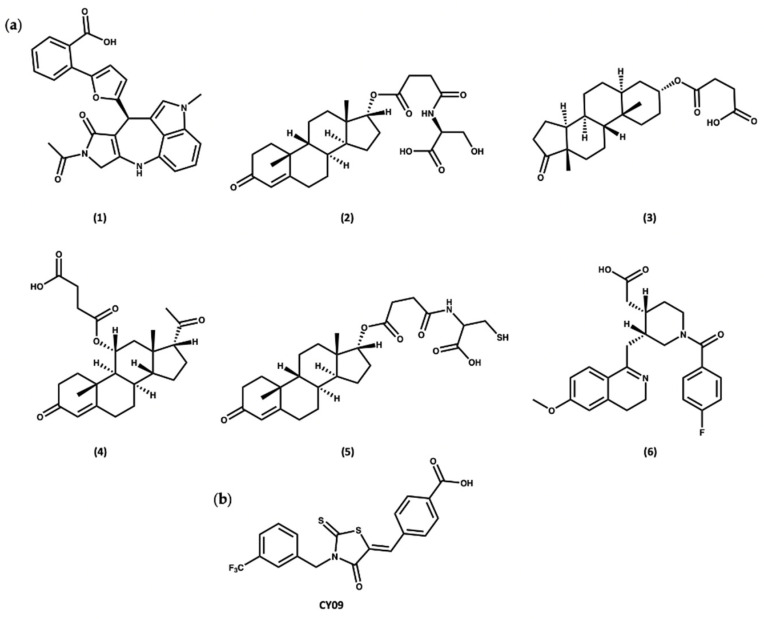
(**a**) Top-ranked compounds predicted from virtual screening of the ZINC20 NP database to bind to the ADP cofactor site of the NLRP3-NACHT structure. (**b**) Chemical structure of CY09.

Docking of the subset from the ZINC20 natural products database in both the cofactor and inhibitor sites resulted in the identification of two sets of compounds. The first group of compounds **1**–**6** showed good binding to the ADP cofactor binding site (Figure 8a and Table S2), which have structural features in common with celastrol. In addition, compounds **1**–**6** all contain a charged carboxylate group, which is predicted to facilitate binding to the Walker A site, similar to the known NLRP3 inhibitor, CY09 (Figure 8b) [88]. The docking scores for compounds **1**–**6** ranged from −16.0 to −17.5, similar to the redocked score of −16.3 for ADP in its X-ray pose and better than the docking score of −14.3 for CY09 (Table S2). 

**Figure 9:**
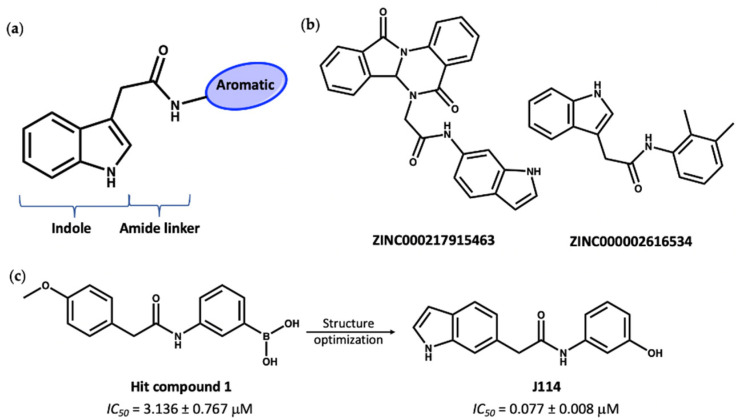
(**a**) General structure of top-ranked compounds predicted from virtual screening to bind to the MCC950 inhibitor site of the NLRP3-NACHT structure. (**b**) Structures of compounds **7** (ZINC ID: ZINC000217915463) and **8** (ZINC ID: ZINC000002616534). (**c**) The reported [91] hit compound **1** and the synthetic active NLRP3 inhibitor, **J114**.

The second group of compounds, ranking top from virtual screening into the known MCC950 ligand binding site, all have a general structure containing an indole ring connected to an aromatic ring through an amide linker; this pharmacophore is denoted in Figure 9a. The indole moiety is a common scaffold in drug discovery, both in synthetic compounds and natural indole alkaloids, with various pharmacological activities [89,90]. The docking scores of this group in the inhibitor site were approximately −14, compared to values of −11.2 and −12.7 for the known inhibitors NP3-146 and MCC950, respectively. Interestingly, these indole compounds, such as **7** and **8** (Figure 9b), have some structural similarity to the recently published compound **J114** (Figure 9c). The latter compound was reported to show both NLRP3 and AIM2 inhibition via inhibition of the interaction between ASC protein and the inflammasome [91]. Yan and coworkers [91] designed compound **J114** (*IC_50_* = 0.07 μM) by structure optimization of the hit compound **1** (*IC_50_* = 3.1 μM) obtained from high-throughput screening (Figure 9c).

## 4. Conclusions 

Non-steroidal anti-inflammatory drugs (NSAIDs), colchicine, and corticosteroids are the most common approaches to the treatment of acute attacks of gout (British National Formulary (BNF) [92]. Xanthine oxidase inhibitors, for example, allopurinol, are used for the long-term control of gout, which decreases the concentration of uric acid, preventing urate deposition (BNF). According to the American College of Rheumatology guidelines and the BNF, IL-1β inhibitors (e.g., canakinumab) could be used for certain cases of gouty arthritis that cannot be treated with NSAIDs [93]. The biologic canakinumab and other IL-1β inhibitors are orally inactive, so it is urgent to design small orally active leads that inhibit proteins involved in IL-1β release. The NLRP3 inflammasome is a cytoplasmic protein complex that is implicated in IL-1β release and inflammation in various inflammatory diseases, including gout. Many studies have revealed that NPs can directly inhibit NLRP3 activity by binding to the NLRP3^NACHT^ domain (e.g., oridonin) or to NLRP3^PYD^ (e.g., β-carotene). Additionally, NPs can inhibit NLRP3 indirectly through blocking of NLRP3-ASC binding (e.g., ILG, CAPE), inhibition of ASC oligomerization (e.g., celastrol), or inhibition of the NF-κB signaling pathway (β-caryophyllene, curcumin). 

To obtain further insights into the preferred scaffolds, we used virtual screening with the ZINC20 database of NPs, resulting in two sets of predicted inhibitors targeting NLRP3. The first group is similar in structure to a known NLRP3 inhibitor either from a natural source (e.g., celastrol), which have a common steroid structure similar to most of the compounds in the first group, or synthetically derived (e.g., CY09) in which the carboxylate group is key to its binding to the Walker A site; these potential inhibitors are predicted to interact with NLRP3 through direct binding to the ADP/ATP site. The second group of compounds, all containing indole rings, have very promising binding energies to the inhibitor site of the NACHT domain. This survey of existing natural product inhibitors of the inflammasome, combined with virtual screening for preferred NP scaffolds targeting NLRP3, highlights the possibilities for the design of novel selective NLRP3 inhibitors inspired by natural products.

## Data Availability

The datasets generated and analyzed in the current study are available from the corresponding author upon reasonable request.

## Supplementary Materials

The following supporting information can be downloaded at: https://www.mdpi.com/article/10.3390/molecules27196213/s1, Table S1: Filter parameters used to filter ZINC20 natural products subset; Table S2: Docking score and similarity score of the best compounds that bind to the cofactor site (compounds **1**–**6**) using the structure of the NACHT domain of NLRP3 (PDB code 7ALV) [35].
