# Supplementary material for: A Selective Review and Virtual Screening Analysis of Natural Product Inhibitors of the NLRP3 Inflammasome
Source: Molecules. 2022 Sep 21;27(19):6213. doi: 10.3390/molecules27196213 (PMC9573361; doi:10.3390/molecules27196213)
Supplement: Supplementary file 1 [file molecules-27-06213-s001.zip › molecules-1908760-supplementary.pdf]

molecules

Supplementary Material

A selective Review and Virtual Screening Analysis of Natural Product Inhibitors of the NLRP3 Inflammasome

Sherihan El-Sayed [1,2], Sally Freeman [1,*] and Richard A. Bryce [1]

Table S1. Filter parameters used to filter ZINC20 natural products subset.

Parameter	Minimum value	Maximum value
Molecular weight	130	550
Number of hydrogen-bond acceptors	0	10
Number of hydrogen-bond donors	0	5
Number of oxygen & nitrogen atoms	0	10
Number O & N atoms with hydrogens	0	5
XLogP	2	5.5

Table S2. Docking score of the best compounds that bind to the cofactor site (compounds **1-6**) using the structure of the NACHT domain of NLRP3 (PDB code 7ALV)[35].

Number	Zinc ID	Chemguass4 score
1	ZINC000604405316	-17.5
2	ZINC000253500112	-17.2
3	ZINC000253500236	-17.1
4	ZINC000253499536	-17.3
5	ZINC000253499916	-16.0
6	ZINC000049590453	-16.7
